# Study on the resin infusion process based on automated fiber placement fabricated dry fiber preform

**DOI:** 10.1038/s41598-019-43982-1

**Published:** 2019-05-15

**Authors:** Ya-Nan Liu, Chongxin Yuan, Chenxiao Liu, Jie Pan, Qinghai Dong

**Affiliations:** Beijing Aeronautical Science & Technology Research Institute of COMAC, Beijing Key Laboratory of Civil Aircraft Structures and Composite Materials, 102211 Beijing, China

**Keywords:** Composites, Aerospace engineering

## Abstract

In this paper, a combination process of automated fiber placement (AFP) of dry fiber and vacuum assistant resin infusion (VARI) was developed, that is firstly a dry fiber preform was fabricated by AFP, and then following a VARI and oven curing process to manufacture the final composite structures, which is expected to be an alternative proposal to the traditional autoclave process, thus making the manufacturing of high performance out-of-autoclave composite structures possible. This research devotes to analyze the character of AFP fabricated preform and its influence on the resin infusion process and the obtained composite. Firstly, the features of dry fiber tapes were analyzed, and a structure model was proposed to illustrate the key factors and issues during the coupled AFP/LCM process. Secondly, the effects of AFP fabricated preform on the resin infusion process and composite performance were analyzed by comparing with manually layup competitors using the same epoxy resin system. It has been demonstrated that the AFP processed preform helps to decrease the porosity from 2.00% to 0.6% and coefficient of thickness variation from 8.11% to 3.75% while improve the fiber volume fraction from 47.80% to 56.30%, however, it prolongs the injection time by 107.71% in this experiment due to lower permeability. Reasonable design of resin flow path and precise control of resin dosage are important for a successful resin infusion process based on AFP fabricated preform. The research can provide preliminary data accumulation and technical basis for further research and future application of AFP based LCM technology in airplane structures.

## Introduction

Advanced composite materials, with light weight, high strength and high modulus, high corrosion resistance and other excellent performances, have been widely used in aero-space structures. In the field of civil aviation, the usage of composite materials has become an important index of the advanced nature of one aircraft^1–3^. However, the widespread applications of composite materials in aircrafts have been greatly restricted by its high cost of manufacture process, which results in high equipment cost and energy consuming in the present autoclave process. Meanwhile, the challenges of interlaminar delamination of composite structures induced by process parameters during aerospace composite manufacture and bonding procedures still remains as an important issue^4–6^. Therefore, light weight, high structure efficiency and low cost composite manufacture technology has attracted much attention in recent years.

Automatic placement technology and liquid composite molding (LCM) technology are both vastly developed in low-cost composite manufacture techniques these years. Compared with handing lay-up methods, automatic placement technology exhibits obvious advantages in higher efficiency, better laying accuracy and more reliable laying quality^7–9^. Automated fiber placement (AFP) technology was developed based on filament winding and automatic tape laying (ATL), which places a number of separate narrowband tows on AFP tooling or mandrels in an automatic fashion to form composite layups or preforms. The material used for AFP process could be thermoset or thermoplastic prepregs, as well as dry fiber tapes. This technology allows better precision and increased deposition rates and could be adapted to those structures with large curvature, thus has gained much attention in advanced industrial areas such as aerospace and aircrafts^10–13^. Early application of AFP technology in large aircraft was the manufacture of engine inlet fairing on B747 and B767. Nowadays, the manufacture of A350 fuselage represents the newest progress of the automatic placement technology, with up to 92 percentage of its fuselage and spars manufactured using AFP technology. Moreover, as the development of AFP technology and the emergence of AFP/ATL common machine, the manufacture of aircraft wing skin was gradually transferred from ATL to AFP process^14–17^. For new generations of future aircrafts, AFP technology is becoming the most preferred method for composite fuselage manufacturing.

LCM is another low cost manufacture method, which using resins infusion and oven curing technology to fabricate composites. LCM process has drawn much attention due to its advantages of less equipment cost, lower energy-consumption, easily for integral molding and less pollution to the environment^18,19^. However, handing lay-up based LCM process always exhibits the disadvantages of low efficiency, poor reproducibility and difficulty in quality control. In addition, as only one vacuum pressure was used during typical vacuum assistant resin infusion (VARI) process, the obtained composites always resulted in low fiber volume fraction and high porosity. Finally, the performance of obtained composite was far from the level equal to that of autoclave composite structure. Therefore, the applications of LCM process in aviation have long been restricted to those functional or sub bearing structures rather than primary structures^18–20^.

Automated dry fiber placement (ADFP) based LCM technology is a combination of AFP technology and resin infusion process, in which dry fiber preforms were firstly prepared by AFP and then composite structures were fabricated using resin infusion and oven curing. The ADFP preform is expected to break the bottlenecks of low fiber fraction in handing lay-up LCM methods through compaction during AFP process, thus making the manufacturing of high-quality composite structure out-of-autoclave with equivalent performance to that of autoclave composite material possible^21–23^. In recent years, the ADFP based resin infusion technology has aroused great attention in aviation industry. For example, Russian MS21 has successful adapted LCM composite to critical primary structures, including its wing spars, wing skins and six section panels for the center wing-box^24–27^. However, the relevant researches mainly focused on the AFP process of preform preparation, while the key process parameters for quality control during resin infusion were not reported. Especially, the features of ADFP preforms and its influence on the resin infusion process and the final composite properties were not clarify yet. Therefore, it is of great importance to conduct researches on ADFP preform based LCM process to solve these basic key problems. Investigation of ADFP preform features and optimization of the following resin infusion process are in much urgent to improve the level of manufacturing capability of advanced low-cost composite materials, and promote further application of LCM technology in primary structures of civil aircraft^28–31^.

In this paper, a combination process of resin infusion and oven curing process basing on AFP process was developed and researched. Firstly, the structure character of dry fiber tapes for ADFP perform preparation was analyzed using SEM, and a structure model was proposed to illustrate the key factors and issues that affect the automatic preform placement and resin injection process. Secondly, the influence of ADFP preform on the resin infusion process was investigated by comparing with that of manually lay-up VARI process. Meanwhile, the obtained laminate quality, including surface quality, thickness uniformity and interlayer morphology, were investigated and analyzed. At last, fiber contents and porosities were tested and discussed using non-destructive test (NDT) and metallographic microscope methods. The obtained results could provide preliminary data accumulation and technical basis for further research and future application of ADFP based LCM technology in aerospace structures.

## Experimental

### Materials and apparatus

AFP dry fiber tapes (PRISM TX1100 dry tape with low epoxy resin content, Cytec), Unidirectional carbon fabrics (HFW160PA, using aerospace grade carbon fiber, Jiangsu Hengshen Co. ltd, China), Epoxy resin (PRISM EP2400 resin system, Cytec, a single part, 180 °C curing, toughened epoxy system); vacuum bags, flowing media, peel ply and other assistant materials (Airtech, the United States).

AFP machine, Coriolis Composites Technologies SAS; Vacuum liquid molding equipment, Isojet Equipments, Sino Composites CO., Ltd; Curing furnace, Etuves, France; Waterjet Cutting System, Flow Waterjet Hong Kong Limited; Scanning Acoustic Microscope, SAM 300E, PVA TePla Analytical Systems GmbH; Field Emission Scanning Electron Microscopy System (SEM), JSM-7100F, Japan JEOL; Thickness gauge, Minitest 7400FH, EPK ElektroPhysik.

### Preparation of composite laminate

**Figure 1:**
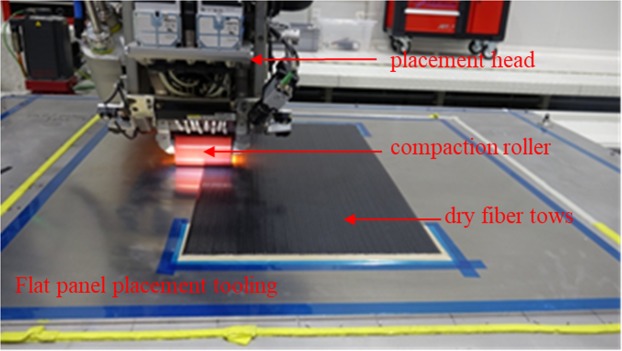
Automated placement process of dry fiber preform using AFP machine.

In this paper, a unidirectional composite laminate was fabricated by ADFP preform based VARI process. Firstly, a unidirectional 6 plies dry fiber preform was prepared using AFP machine, which adapted to layup of 16 tows dry fiber tapes in thickness of 0.1 mm to 0.3 mm and width of 6.35 mm. Figure 1 shows a picture of AFP processing dry fiber preform procedure. A laser heating head was adapted for the heating and compression of dry fiber tapes during placement. The commonly used default settings of AFP process parameters were used, and the temperature of paving area was monitored and tested to be about 160 °C during placement process at a placement speed of 200 mm/s. Meanwhile, a compaction system was used to ensure that each fiber can be compacted in the placement surface, and a compaction press of 160 N was used here.

**Figure 2:**
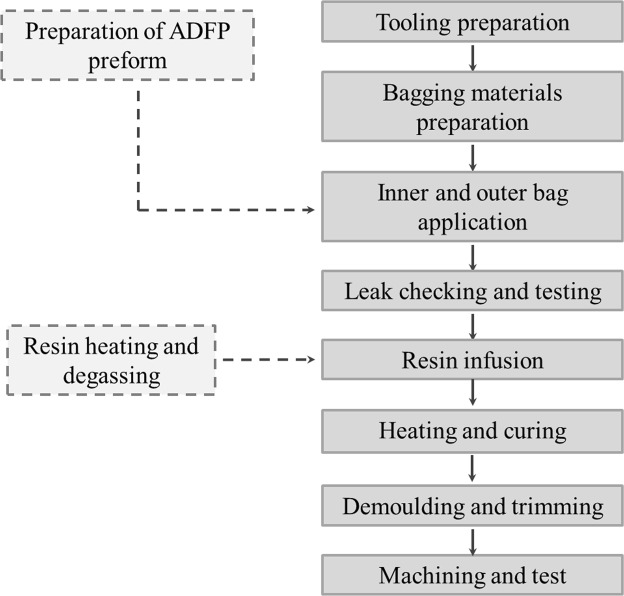
The flow chart of the preparing procedure for ADFP preforms based resin infusion process.

After preparation of dry fiber preform, a vacuum assisted resin infusion and oven curing process was used to fabricate the final composite laminate. Figure 2 illustrates the flow chart of the preparing procedure. Firstly, a flat infusion tooling was cleaned and leak checked, and a kind of mold release agent was used to the mold surface before layup of bagging materials. Then, the bagging materials such as vacuum bag, flow media, release film, peel ply and seal tapes were prepared.

**Figure 3:**
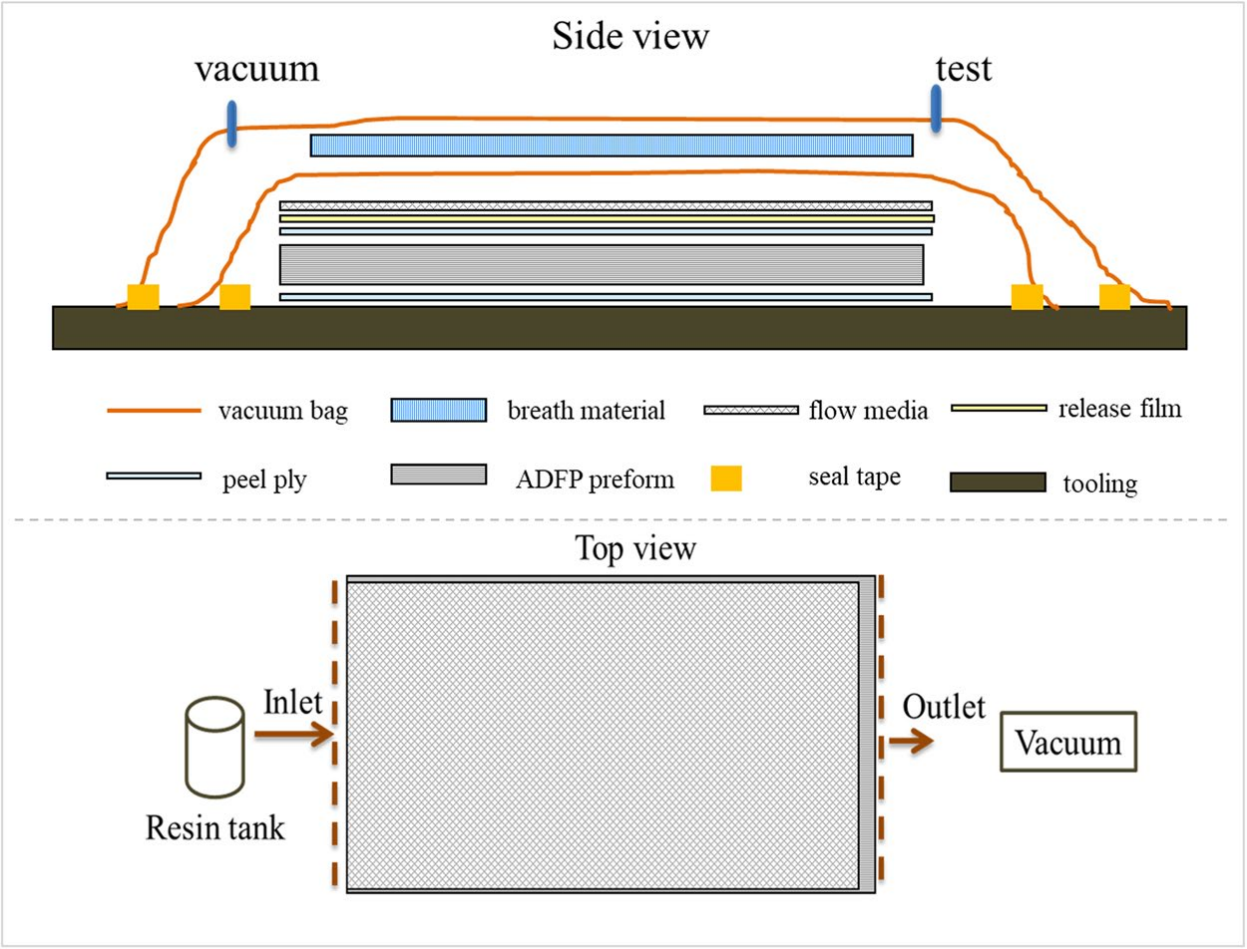
The schematic diagram of vacuum bagging layouts used for resin injection.

After prepared, a double layer vacuum bag systems was applied as shown in Fig. 3, which following by leak checking and testing. At the same time, the resin system was heated and degassed in vacuum oven. Finally, the completed setup was transferred to a curing oven, in which the degassed resin was infused into the preform from one edge of the preform to the other end, and the flow path was illustrated in the top view of Fig. 3. After injection, the system was cured according to an aerospace standard curing cycle of 180 °C for 120 minutes. After the curing process, the setup was naturally cooled down to room temperature and demolded. At last, the obtained laminated was trimmed and machined using Waterjet Cutting System for performance testing and analyzing.

For comparison, another composite laminate was prepared by manually lay-up VARI process, in which a unidirectional carbon fiber cloth was chosen as raw materials, while other conditions and parameters were controlled as the same as that of the ADFP preform based VARI process.

### Testing and analysis

The thickness of the ADFP preform and the final laminates were measured by the thickness gauge. The surface and fracture morphology of AFP fiber tape and obtained composite laminates were observed by a SEM. The defects and inner quality of the laminates were investigated by Scanning Acoustic Microscope. The composite porosity was tested using metallographic microscope according to GB3365.

## Results and Discussion

### Characterization of ADFP preform

**Figure 4:**
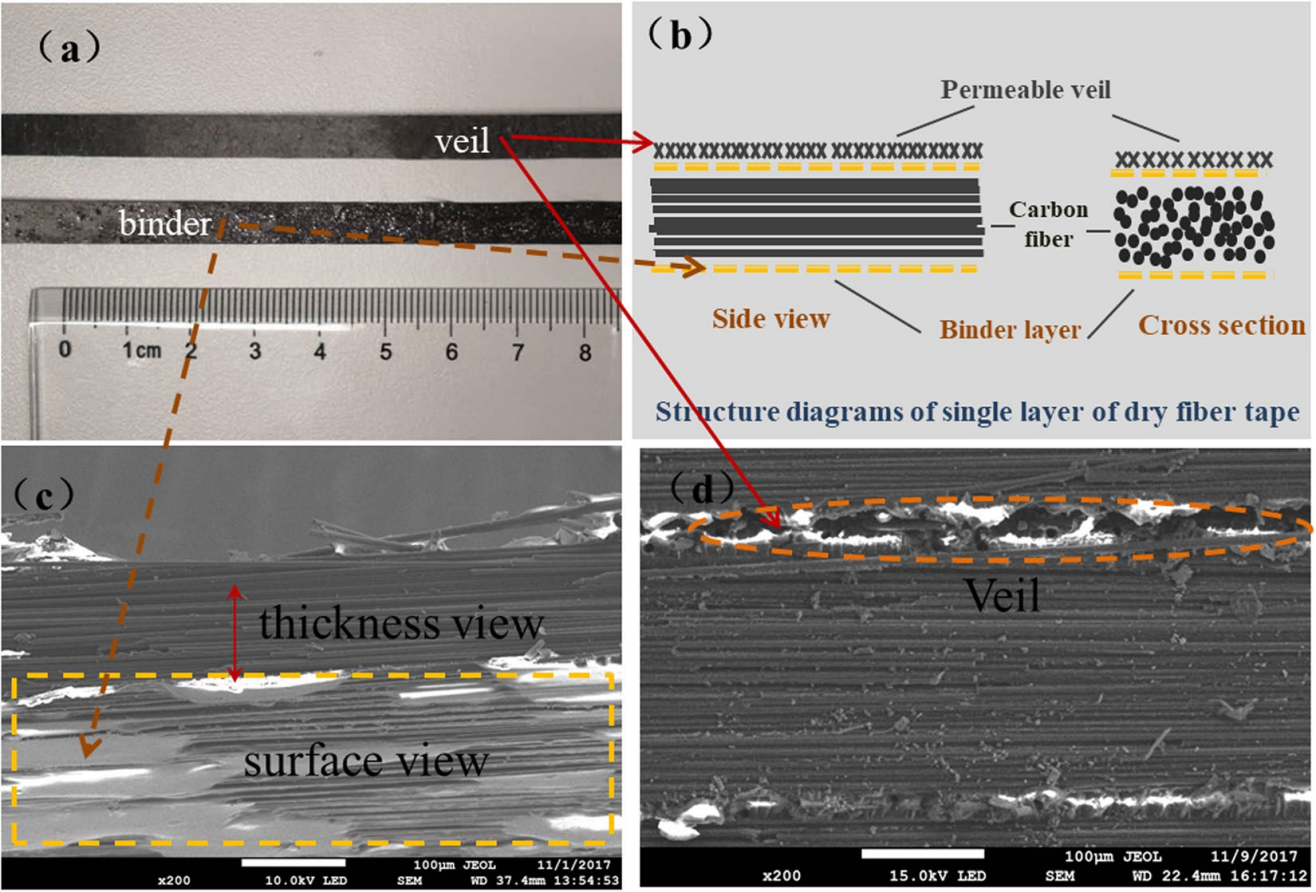
The surface photos and structure diagrams of single layer of dry fiber tape and SEM images of its microscale morphology. (**a**) Surface of dry fiber tape, (**b**) structure diagrams of single layer for dry fiber tape, (**c**) surface and side view SEM images of single fiber layer, (**d**) inter layer SEM images parallel with fiber direction.

In this paper, a kind of dry fiber tapes TX1100 with very low resin content was used as raw material to prepare the preform during automatic fiber placement procedure. The dry fiber tape is in thickness of 0.1 mm to 0.3 mm and in width of 6.35 mm. Figure 4(a) shows a photo of a small cut of the tape. It can be seen that the surface of one side is rough and black, while the surface of the other side is smoother with some flash pots. To analyze the character of ADFP preform, a structure model was proposed to illustrate the single-layer structure of dry fiber tape, as shown in Fig. 4(b). In this model, the dry fiber tape was believed to be a heterogeneous structure rather than a uniform structure. The heterogeneous structure with complex sub structures should simultaneously conform to the requirements during AFP process and the subsequent resin infusion process.

On the one hand, the dry fiber tape should meet the requirements during automatic laying process. Firstly, the 6.35 mm width narrowband tape exhibited outstanding turning and pavement ability, which greatly benefited the AFP process for satisfactory and accurate placement comparing to those 150 mm or 300 mm width ATL tapes, especially for curve and complex structures. Secondly, the flash pots on the smoother surface of Fig. 4(a) is a thin resin layer, named binder, which can be proved by the SEM images in Fig. 4(c). The binder layer, working as fixing and adhesive agent during AFP procedure, provides enough tack to affix each layer and maintain the shape and integrity of the preform. During AFP process, a laser heating was adopted and used to heat the resin in binder layer, which became tacky and bonded all the fiber layers together and finally formed and kept the shape of preform. However, too much tack would be also another problem. It is of great importance that the binder should be separately dispersed on the tape surface rather than completely coated, as shown in Fig. 4(a,c). In fact, we might recommend that the adhesive binder spots be distributed in a network on the tape surface. Furthermore, the binder resin contains a molecular structure that is compatible with the EP2400 resin system used in the following resin infusion process. Thus, binders can blend well with the resin matrix after curing, without affecting its performance.

**Figure 5:**
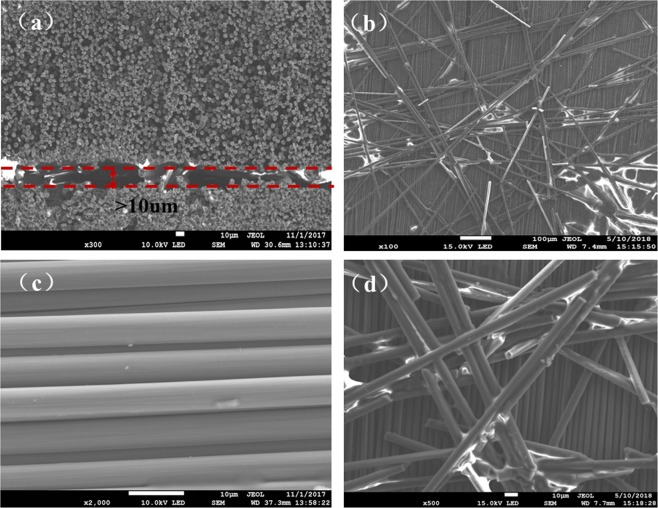
(**a**) Inter layer SEM images vertical to fiber direction, (**b**) SEM images of veil layer, (**c**) SEM images of continuous fibers, (**d**) network structures of the veil layer.

On the other hand, the post resin infusion process and final composite quality are greatly depended on the preform permeability. To get satisfactory surface and inner quality of final composite parts, it is necessary to ensure completely infiltrating during resin infusion and appropriate fiber/resin interfacial adhesive strength after curing. Thus, the dry fiber tape should also meet the requirements during resin injection process. The rough surface photos in Fig. 4(a) and interlayer SEM images of Fig. 4(d) demonstrated the existence of rough veil layer, which helps to improve the permeability of preform during resin infusion. To analyze the modification effect of veil layer on preform permeability, further evidence was obtained by observing the structure of veil layer. As shown in Fig. 5(a,b), the rough black veil layer is about 10um in thickness, and made of randomly distributed chopped carbon fiber mat. Figure 5(d) further demonstrated that a loosely high porosity network structure was formed by short fibers for veil layer. It is obvious that resin permeability would be much easier through this kind of network structure, comparing to the neat structure formed by densely packed unidirectional long fibers (Fig. 5(c)). Thus, the veil layer exists as a high permeability flow media, facilitating the process of resin penetrating into the ADFP preform.

In addition, proper fiber/resin interfacial bonding property was also an important factor for satisfactory composite property. As can be seen in the fiber surface morphology of Fig. 5(c), there are visible grooves in the surface of the carbon fiber, which can provide mechanical engagement sites during fiber/resin infiltration process and help to improve the fiber/resin interface bonding strength. Thus, it is clear that the multiscale structure model (Fig. 4b) proposed was demonstrated to have the combination advantages for both preform preparation and resin infusion processes.

### Influence of ADFP preform on resin injection process

**Figure 6:**
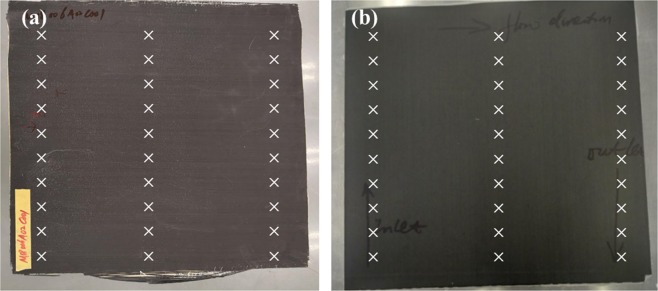
The thickness test points of (**a**) manually and (**b**) ADFP based laminates.

Both ADFP preform based VARI process and manually lay-up VARI process were used to fabricate composite laminates as described in section 2.2. For the sake of convenience in comparison, we denoted them as ADFP and manually for short, respectively. The dimensions for both ADFP and manually lay-up based composite panels were kept the same as 500 mm by 500 mm, as shown in Fig. 6. Herein, the thickness and resin infusion process were compared and investigated.

The thicknesses before and after curing of the two counterparts were tested using a digital thickness gauge. Ten points were evenly taken on the inlet side, the outlet side and the middle, with totally 30 test points taken for each preform and laminate. The thickness test points were illustrated in Fig. 6 and the average thickness results were concluded in Table 1.

**Table 1:** The thickness results analysis of different composite laminates (mm).

	preform			laminate		Thickness change rate (%)
	Thickness	Cv (%)	Weight	Thickness (mm)	Cv (%)	
manual	1.556 ± 0.05	8.51	240	1.194 ± 0.198	8.11	−23.26
ADFP	1.21 ± 0.03	2.48	226.5	1.283 ± 0.061	3.75	6.03

Benefiting from the compact force applied to each layer during AFP process, the ADFP preform was more tightly stacked and more uniform in thickness, comparing to the situation in manually lay-up composite laminate. The laser heater and the compacting roller contributed to the tightly packed structure of preform. It can be seen form Table 1 that the thickness uniformity of final composites was improved for ADFP preform comparing to its manually prepared competitor. The coefficients of thickness variation for ADFP based composite were 2.48% and 3.75% before and after curing, while the manually prepared composites showed much higher values of 8.51% and 8.11%, respectively.

The increase in thickness uniformity of ADFP system mainly resulted from two reasons. Firstly, as discussed in section 3.1, the dry fiber preform prepared by AFP has the advantages of higher compacted structure due to laser heating and compact roller press, which helps to modify the uniformity in thickness. Secondly, a double vacuum bagging system was adopted in this paper, the inner bag provided the infusion channel and infiltration force for resin flow, while the outer vacuum bag formed a uniform pressure on the top surface of the laminate, and simultaneously ensured that the vacuum level meets requirements during heating and curing procedures. Thus, both the well and tightly packed preform and the double vacuum bag system contributed to improve the thickness uniformity and surface quality of ADFP composite.

Furthermore, an index of thickness change rate was defined in this paper to illustrate the thickness change before and after curing. As shown in equation 1, thickness change is the percentage of thickness variation after curing in related to the preform thickness before curing, where T2 is laminate thickness after curing and T1 is preform thickness before curing. The thickness change rate of two different laminates was calculated using the following formula, with the results shown in Table 1. It can be seen that the manually lay-up and ADFP based laminates showed different thickness change rates of −23.26% and 6.03%, respectively. It is obvious that the thickness variation is much smaller for ADFP system than that of manually lay-up system, demonstrating that ADFP based VARI process is more convenient and adapt to accurate and precise control for part shape and dimension. In addition, we notice that the thickness change value is positive for ADFP composites, while the manually lay-up system exhibited a negative value. The small increase in ADFP thickness after curing might result from the introduction of infused resin. However, the obviously decreased thickness of manually lay-up system might mainly because of the low density of the manually lay-up layers. The vacuum pressure and the surface tension during resin injection process have greatly pressed the loosely packed dry fiber layers.

1 $${\rm{Thickness}}\,{\rm{change}}\,{\rm{rate}}=({\rm{T}}2-{\rm{T}}1)\times 100 \% /{\rm{T}}1$$

Furthermore, effects of ADFP preform on the resin dosage and injection time were also investigated. As the other parameters kept the same, the amount of resin infused and injection time of the two processes was compared, with the results shown in Table 2. It can be seen that the infused resin weight in manually lay-up system is higher than that of ADFP process, while the injection time for ADFP based process is 107.71% longer than that of manually lay-up process in this experiment. Less injection resin weight indicated that the content of resin fraction in composites is lower, and correspondingly the fiber content is higher. Higher fiber content would be critical to improve the composite performance, revealing the advantages of ADFP based VARI process comparing to manually lay-up process. The obvious prolonged injection time was also due to the densely packed structure of the ADFP preform, leading to lower permeability and difficulty in resin infiltration. Therefore, in the development of this coupled AFP/resin infusion process in real aricraft composite structures, the injection time and fiber content should be well designed and balanced.

**Table 2:** comparison of resin injection process.

	Infused resin weight (g)	Injection time (min)
manual	440	35
ADFP	389	72.7

### Composite laminate quality analysis

**Figure 7:**
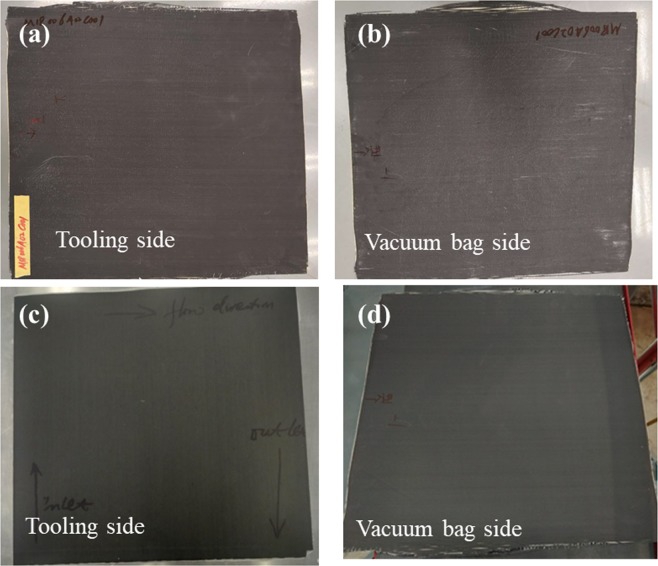
The surface photos of obtained laminates. (**a**,**b**) manually layup, (**c**,**b**) ADFP preformed.

After comparison of resin injection process, the obtained composite laminate surface and internal quality were further analyzed. Figure 7 shows surface photos of obtained laminates by ADFP based and manually lay- up resin infusion process, with the same dimensions of 500 mm by 500 mm. As can be seen from Fig. 7, the surfaces opposite to the isnfusion tooling of both two laminates were flat ad well infiltrated, without visible dry islands or other defects. However, difference was shown between two laminates on the vacuum bad side surfaces. It can be notice in Fig. 7(b,d) that the vacuum bad side surface for ADFP based laminates was much better than that of manually lay-up laminates. The situation agrees well with the thickness variation results in Table 1.

**Figure 8:**
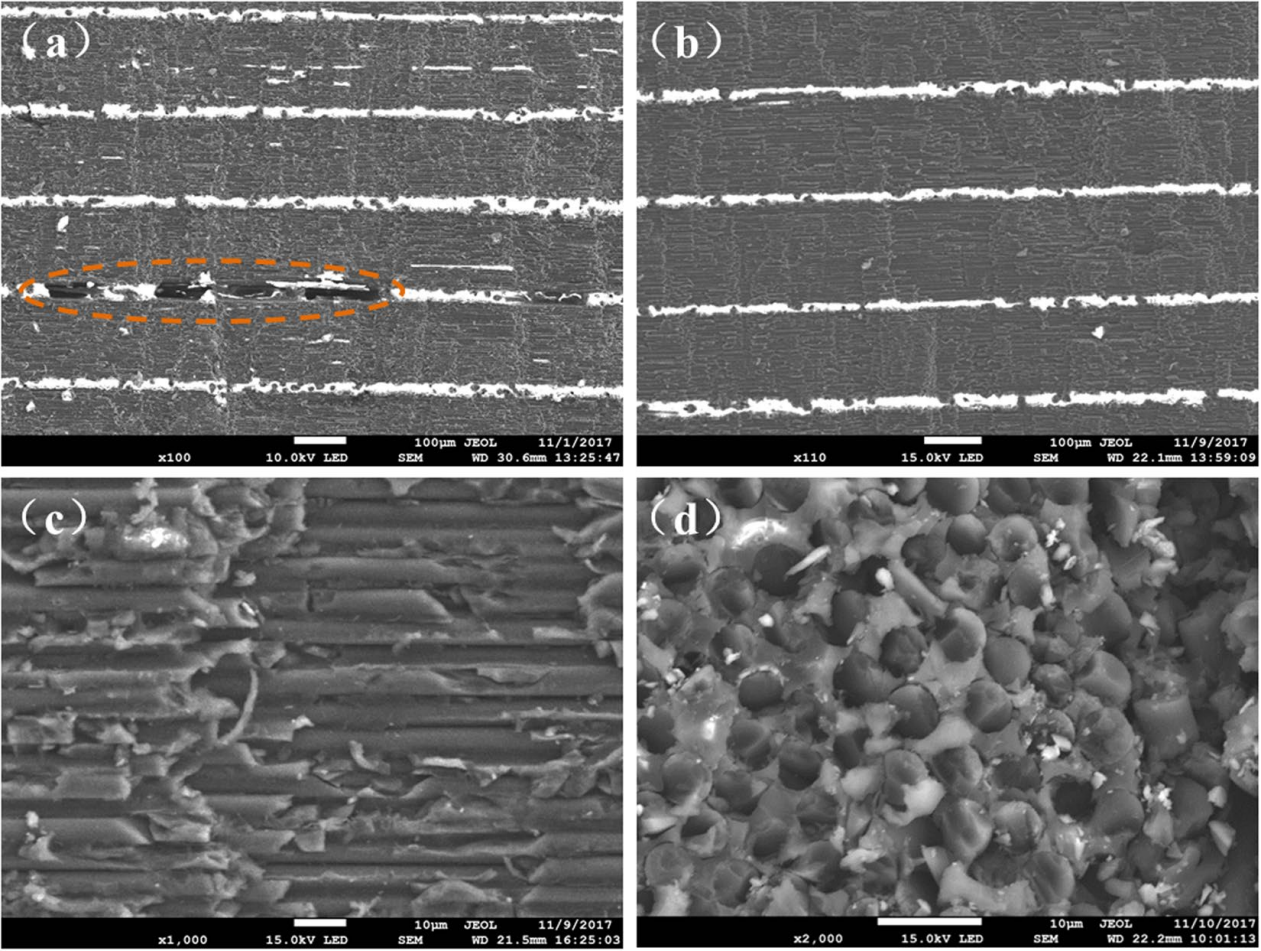
SEM images of interlaminar morphology of ADFP based resin infused laminate.

In addition, the thickness uniformity of obtained ADFP laminates was also investigated by SEM, as shown in Fig. 8(a,b), it can be seen directly and clearly that the thickness of each layer in side view of the laminates is in good uniformity with an almost straight inter-laminar interface. Benefiting from the compaction force during AFP process, the layer thickness was well controlled and the laminate was densely packed without obvious delamination, wrinkles or porosity.

As discussed above, a laser heating and compaction roller were applied during the preparation of dry fiber preform by AFP technique, aiming to ensure the quality of obtained preform. The compress used in preform preparation can effectively improve the surface flatness and thickness uniformity to get a denser and well packed structure, thereby increasing the fiber content of final composite laminate and ultimately improving the composite properties. However, comparing to handing lay-up preforms, the densely packed structure of ADFP preform also exhibited disadvantages in difficulties of resin penetration into fiber during the subsequent resin injection process. Especially, the resin permeability through thickness direction among different layers is much lower, which intends to forming defects and lead to incomplete infiltration, such as large pores between layers, as shown in Fig. 8(a).

In this paper, a double vacuum bag system was adopted during resin infusion and curing process, and the process parameters were optimized, especially the amount of resin used was precisely calculated and controlled, which helped to ensure the quality of final laminate. As can be seen from Fig. 8(b), the preform was completely penetrated and wetted by resin through the thickness of obtained composite laminate, without obvious porosity, delaminating or other defects. Further observations of the fracture surface in both directions of parallel and perpendicular to fiber orientation were taken by SEM and shown in Fig. 8(c,d), which reveal that the resin matrix penetrated completely into the preform and good fiber/resin interfaces were formed. Good interface could be helpful for effective stress transfer, providing a microstructure basis for the satisfactory performance of ultimate composite laminates or structures.

**Figure 9:**
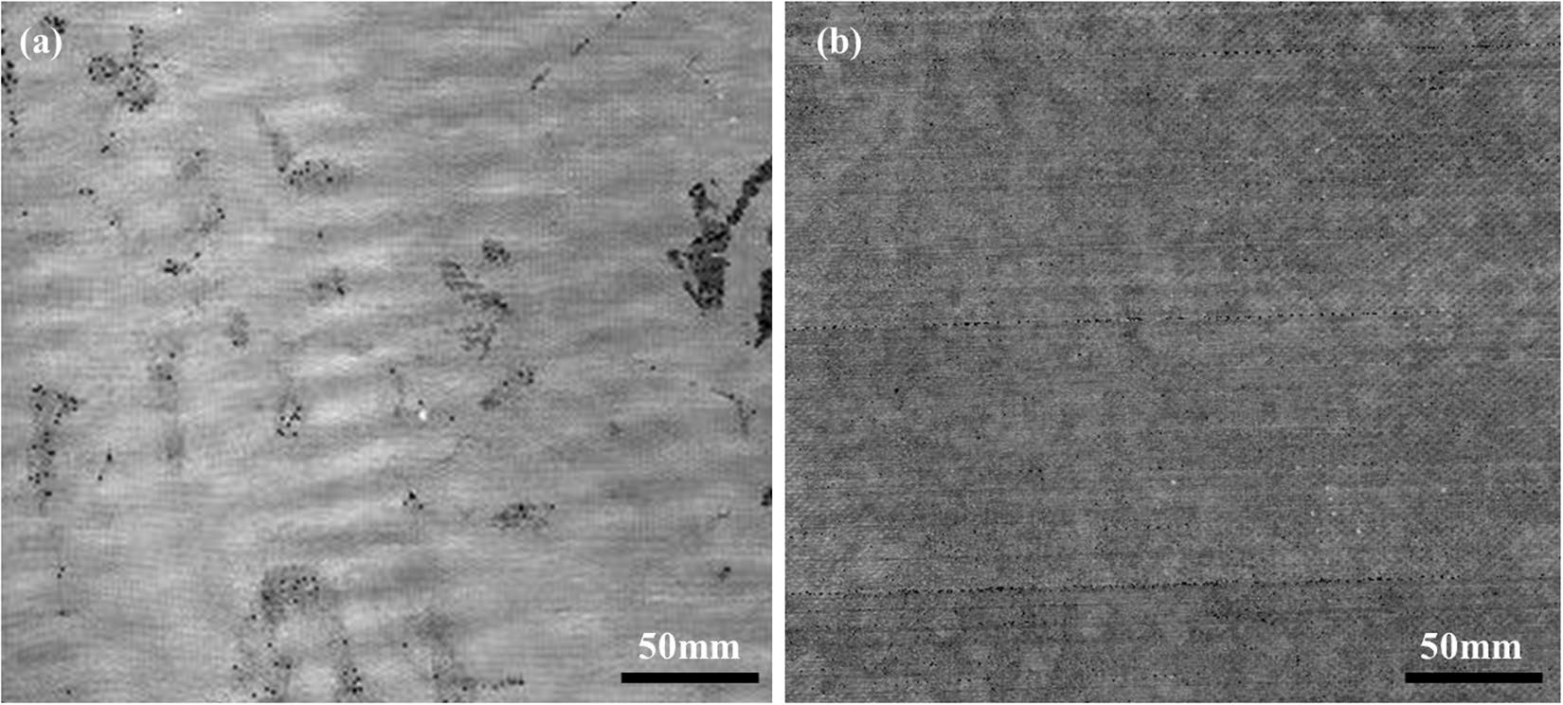
NDT results of (**a**) manual based laminate and (**b**) ADFP preform based laminate.

In order to further analyze the internal quality of the prepared composite laminate using ADFP based resin infusion and oven cured process, NDT was performed using a scanning acoustic microscope. An ultrasonic immersion reflection method was carried out using a scanning acoustic microscope system, with a 5 MHz probe in diameter of 9.5 mm (0.375 inch) and a scanning area of 300 mm by 300 mm, under a focal distance of 20 mm (0.79 inch). Figure 9 shows the typical scanning image of obtained manual and ADFP based resin infusion laminates, respectively. It can be seen from Fig. 9(a) that, there were some black areas in NDT scanning image of manual lay-up laminate, indicating porosity or inclusion defects. The defects in manual lay-up laminate might be resulted from those dropped short cutting fibers during handing lay-up, which was not completely eliminated from layout and finally retained as undesirable inclusions. In contrast, it can be seen from Fig. 9(b) that there was no obvious porosity, delamination, debonding or undesirable inclusions in the ADFP based laminate, agreeing well with the results shown in SEM images in Fig. 8(c,d). Furthermore, the scanning image of NDT test for ADFP based laminate showed uniform in image brightness, indicating that the laminate quality of different sites were uniform, without obvious variation, which once again demonstrated the advantage of ADFP in quality control.

**Figure 10:**
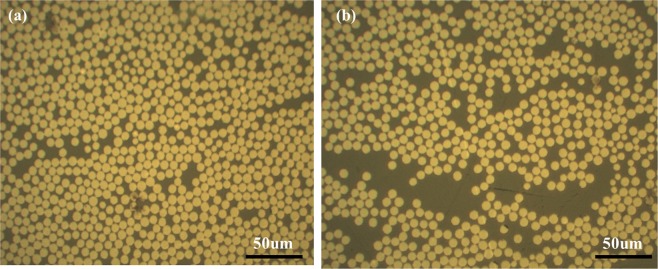
Typical metallographic photos of (**a**) ADFP based laminate and (**b**) manually lay-up laminate.

In addition to NDT results, the fiber volume fraction and porosity of obtained laminates were tested using metallographic microscope according to GB3365. Figure 10 shows the typical metallographic photos of ADFP based and manually lay-up laminates. It demonstrated further that the ADFP base laminates exhibited more densely packed structure than that of handing lay-up laminate. According to test standard, 5 samples from different area were prepared and tested to calculate the fiber content and porosity average values. Finally, the fiber volume fraction was test to be 56.30% for ADFP based laminate and 47.8% for manually lay-up laminate, respectively. As to the porosity, among the 5 test specimens of ADFP laminate, only one of them showed a porosity of 0.60%, while the other four did not show obvious porosity. For the manually lay-up specimens, the porosity was found varied from 1.0% to 2.0%. As discussed above, the structure of dry fiber tapes and the former AFP process helps to improve the composite quality and fiber contents, thus greatly improved the performance and potential application of out-of-autoclave composites in aircrafts.

## Conclusions

In this paper, aiming to improve the application of out-of-autoclave composite structures on civil aircrafts, a combination process of automated fiber placement of dry fiber followed by vacuum assistant resin infusion and oven curing was developed.

Firstly, the structure character of dry fiber tapes was analyzed, and a heterogeneous structure model was proposed and demonstrated. The raw materials need to satisfy the requirements of both AFP and resin injection process. Secondly, the effects of AFP processed dry fiber preform on the following resin infusion process were analyzed by comparing to that of manually layup system, using aerospace grade unidirectional carbon fabric and a toughened epoxy resin system. It has been demonstrated that AFP fabricated dry fiber preform helped to improve the composite quality, resulting in a higher fiber volume fraction up to 56.3%, a less porosity of 0.60% and a better surface flatness with coefficient in thickness variation of 3.75%, comparing to the values of 47.80% in fiber volume fraction, 2% of porosity and coefficient of thickness variation of 8.11% for manually lay-up laminates. However, it should be noticed that the AFP processed preform greatly prolong the injection time by 107.71% in this experiment due to lower permeability. It is of great importance to control satisfactory composite quality by reasonable design of resin flow path and precise control of resin dosage. The obtained results could provide preliminary data accumulation and technical basis for further research and future application of dry fiber AFP with a resin infusion process in airplane structures.
